# A Rare Case of Leukemia Cutis as the First Presentation of a Myelodysplastic Syndrome to Acute Myeloid Leukemia Transformation

**DOI:** 10.7759/cureus.8698

**Published:** 2020-06-19

**Authors:** Dawood Findakly, Surabhi Amar

**Affiliations:** 1 Internal Medicine, Creighton University Arizona Health Education Alliance/Valleywise Health Medical Center, Phoenix, USA; 2 Oncology, Valleywise Health System, Phoenix, USA; 3 Internal Medicine/Hematology and Oncology, University of Arizona, Phoenix, USA

**Keywords:** leukemia cutis, therapy-related myelodysplastic syndrome, acute myeloid leukemia (aml), new insights, cytogenetic analysis

## Abstract

Leukemia cutis (LC) is a rare presentation of leukemia. It is characterized by the infiltration of leukemic cells into the different layers of the skin causing varying skin manifestations. It can occur before the hematological presentation of leukemia or during the disease course and carries a poor prognosis. Here, we report a patient with therapy-related myelodysplastic syndrome (t-MDS) whose transformation to acute leukemia was heralded by the development of LC. Worrisome cutaneous lesions should not be overlooked, and a skin biopsy should be pursued to confirm the diagnosis. A high index of suspicion is the key to early recognition of sometimes nonspecific skin findings of widespread systemic disease.

## Introduction

Leukemia cutis (LC) is the skin infiltration by malignant hematopoietic cells leading to varying dermal manifestations ranging from a simple rash to nodules and patches. Its incident varies in different leukemias but most commonly reported in acute myeloid leukemia (AML), where the incidence ranges from 5% to 10% [1-4]. We report a case of a patient with an acquired immunodeficiency syndrome (AIDS) who was treated for and cured of diffuse large B-cell lymphoma (DLBCL). Three years later, he developed therapy-related myelodysplastic syndrome (t-MDS) and treated with a hypomethylating agent. Ten months into therapy, the development of LC heralded disease transformation to AML.

Here, we emphasize the principal role of clinicopathologic correlation through biopsy from the cutaneous lesions with immunohistochemical (IHC) stains to recognize the malignant infiltrates in hematopoietic malignancies, and highlight the chromosomal aberration found upon cytogenetic analysis.

## Case presentation

**Figure 1:**
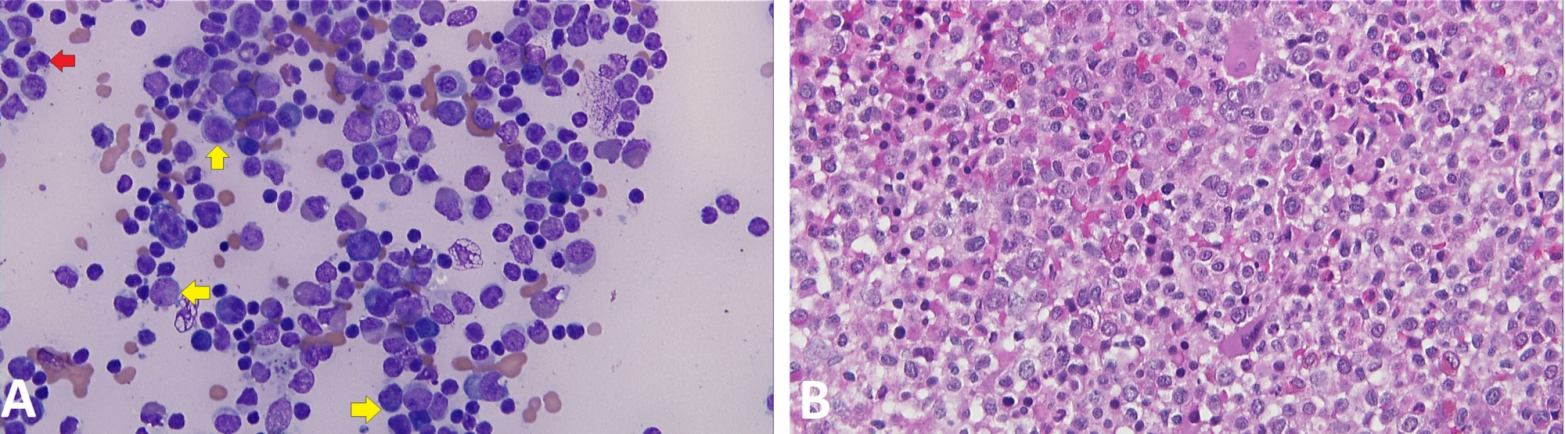
BM aspirate and core biopsy showing myeloid neoplasm (myelodysplastic syndrome) with no increase in blasts. (A) BM aspirate smear showing dysgranulopoiesis with abnormally lobulated and hypogranular neutrophils (red arrow) and less than 5% blasts (yellow arrows). (B) BM core biopsy showing hypercellular BM with greater than 95% cellularity. BM: bone marrow

A 50-year-old-man with a history of AIDS noted to have pancytopenia on a routine visit. Three years ago, he was diagnosed with stage IVB DLBCL and treated with rituximab plus cyclophosphamide, oncovin, and prednisone (RCHOP) regimen, and he had been in complete remission since then. The patient was asymptomatic, and the physical examination was unremarkable. His laboratory workup was relevant for hemoglobin of 7.6 g/dL, platelets of 79 K/µL, white blood cells (WBC) of 3.5 x10^3^/µL with 39% neutrophils, 23% lymphocytes,0% eosinophils, and 13% monocytes with 6% blasts, but flow cytometry did not show any increased blasts. A subsequent bone marrow aspiration and biopsy reported a hypercellular marrow with some evidence of dysplasia (Figure 1).

**Figure 2:**
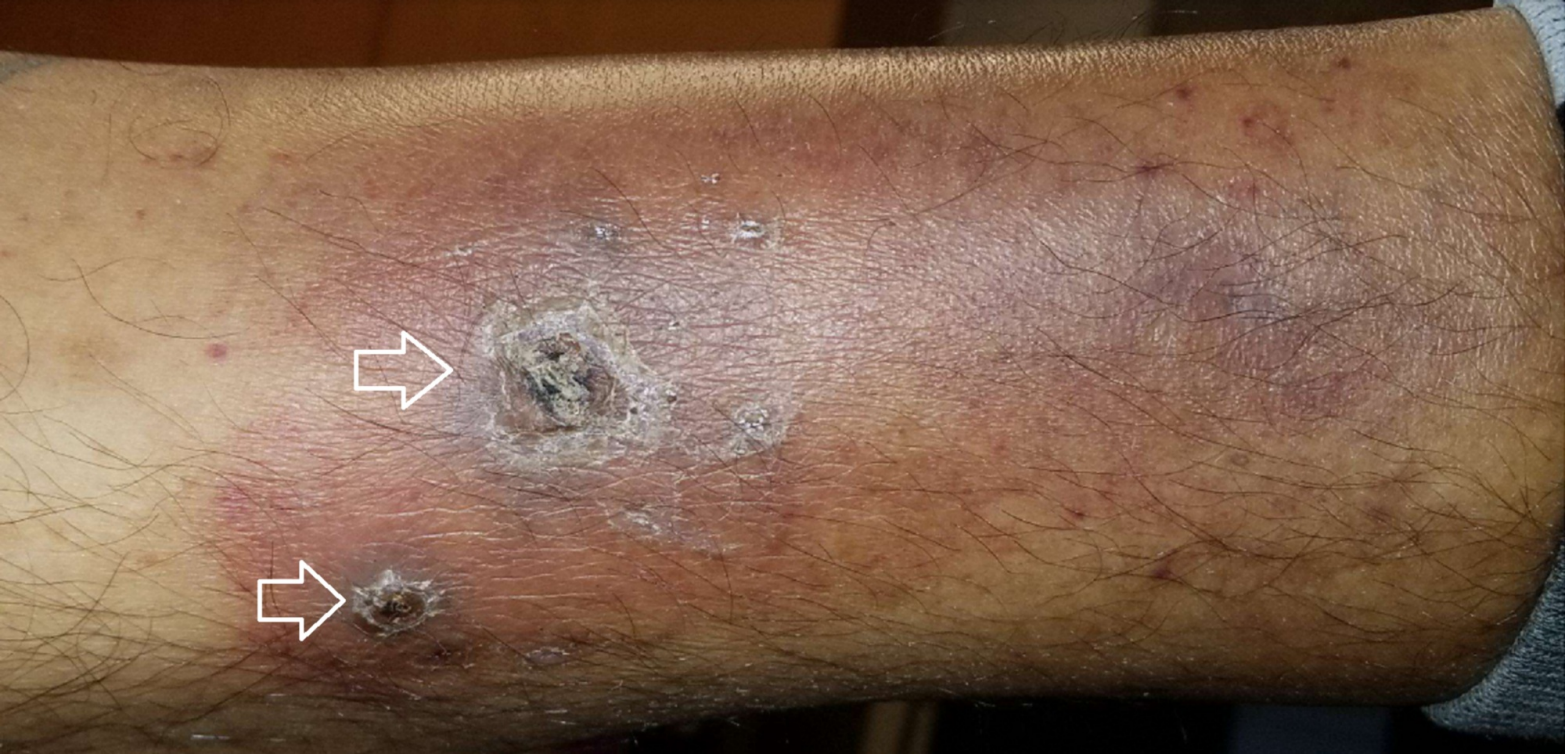
The clinical picture of the lower extremity nodular skin eruptions associated with purpura and crustation (white arrows).

**Figure 3:**
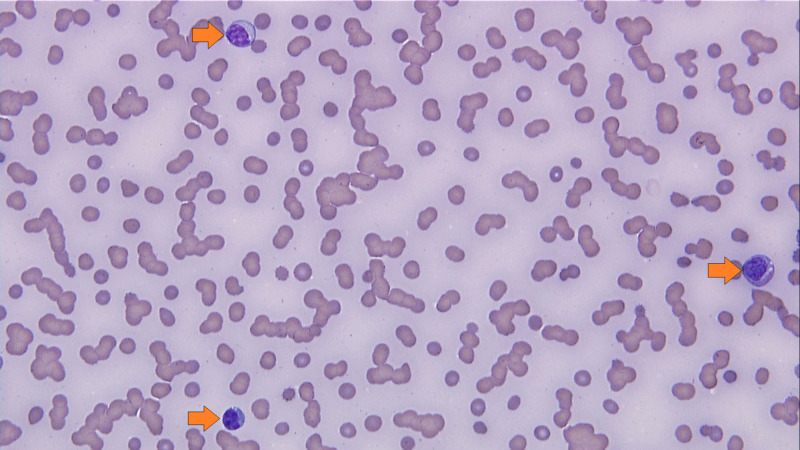
Peripheral blood smear showing occasional blasts comprising around 1% of the total peripheral blood leukocytes (arrows).

The cytogenetics showed 46, XY, and (11)(p15q23); therefore, he was diagnosed with t-MDS and started on azacytidine. The patient remained relatively asymptomatic for 10 months until he was noted to have lower extremity nodular skin eruptions with purpura and crustation (Figure 2), although his blood counts remained stable with 1%-6% blasts on peripheral smear on various occasions (Figure 3).

**Figure 4:**
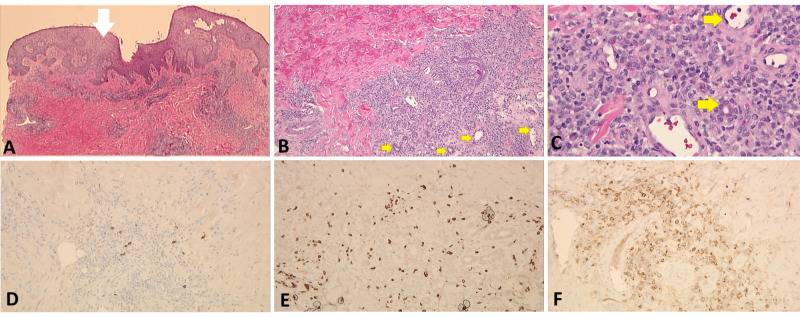
H&E staining performed on the punch biopsy specimen revealing: (A) epidermal spongiosis and mild hyperkeratosis (white arrow) at x20 magnification. Moderately prominent dermal perivascular and peri-eccentric, infiltrate of mononuclear cells with irregularly shaped, vesicular nuclei, and small nucleoli with occasional mitotic figures (yellow arrows) seen at (B) x100 magnification and (C) x400 magnification. Moreover, IHC performed (at x200 magnification) for (D) CD3, (E) CD45, and (F) CD68. H&E: hematoxylin and eosin; IHC: immunohistochemistry; CD: cluster of differentiation

A punch skin biopsy revealed an inflammatory cutaneous infiltration with a population of large, irregularly shaped vesicular nuclei and small nucleoli (Figures 4A-4C). IHC staining was positive for cluster of differentiation (CD)68, CD117, and CD45 and negative for CD3 and CD2 (Figures 4D-4F). The IHC results mentioned above, together with the patient's history of t-MDS, indicated LC as a rare initial presentation of the transformation to AML.

**Figure 5:**
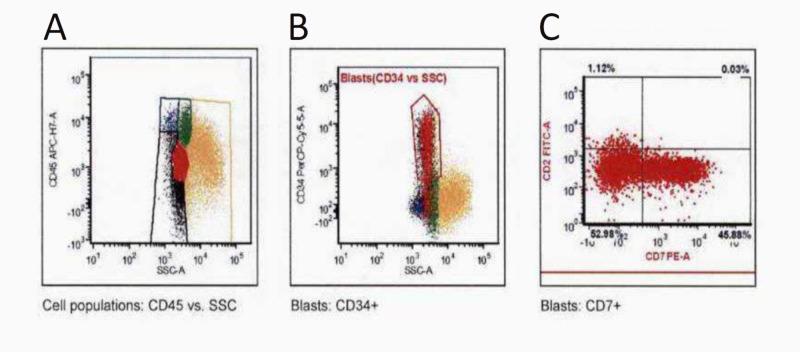
Flow cytometry plots revealing 26.2% myeloid blasts most compatible with treatment-related AML in the setting of MDS. (A) CD45+ (dim) expression; (B) CD34+ expression; and (C) CD7+ (partial) expression. AML: acute myeloid leukemia; MDS: myelodysplastic syndrome; CD: cluster of differentiation

Over the next four weeks, he developed a low-grade fever, bone pains, and anorexia with increasing leukocytosis, worsening anemia, and thrombocytopenia. Laboratory workup showed a hemoglobin of 7.1 g/dL, WBC of 73 x10^3^/µL with 14% neutrophils, 7% lymphocytes, 0% eosinophils, 23% monocytes, 26.1% blasts, and platelets of 21 K/µL. Subsequent flow cytometry showed 26.2% myeloid blasts most compatible with therapy-related AML in the setting of MDS (Figure 5).

Therefore, the option for inpatient treatment with liposomal cytarabine and doxil (daunorubicin) or mylotarg (gemtuzumab ozogamicin) was offered with the caveat of intensified risk of morbidity and associated mortality with the induction therapy. After detailed consideration, the patient decided to forgo aggressive treatments and was provided with supportive care and blood transfusions. We conducted further discussion regarding the terminal and progressive nature of his condition. The palliative care team was involved in the patient's care, and he was referred to hospice, where he eventually died three months after his LC diagnosis.

## Discussion

Leukemias, especially acute leukemias, can present with a spectrum of extramedullary myeloid cell tumors, including skin infiltrates with or without bone marrow disease [4]. LC is a rare neoplastic condition characterized by the extramedullary infiltration of the skin or subcutaneous tissue by leukemic cells. LC could be an incidental finding when leukemic cells are, unexpectedly, identified upon skin lesion's evaluation, making a leukemia diagnosis [5]. More commonly, it is noted in the setting of a previously diagnosed hematologic malignancy, but rarely it may be the first sign of a new leukemia diagnosis [5,6]. The interval from skin biopsy to the diagnosis of systemic leukemia can vary from three weeks to 20 months [7,8]. The appearance of skin lesions is nonspecific and can vary from flesh-colored to purple papules, plaques, or nodules. Prioritizing management to treat leukemia becomes a crucial aspect of the overall patients' survival [5]. LC is often associated with additional locations of extramedullary involvement that forecast a poor prognosis. The median two-year survival for AML patients with LC is <10% compared to 30% in the absence of LC in some studies [6,9]. Given its diverse and nonspecific clinical presentation, LC diagnosis relies exclusively on IHC [10]. The skin biopsy in our patient showed positive IHC for CD45, CD68, and CD117, and negative for CD3 and CD2. Systemic therapy is the mainstay of treatment for AML with LC.

In our patient, LC was the first manifestation of the transformation of t-MDS to acute leukemia and predated hematological manifestation. The clinicopathologic correlation of cutaneous manifestations in patients with pre-existing hematologic malignancy is crucial in recognizing this condition. Interestingly, this patient had the inv(11)(p15q23) chromosomal aberration via cytogenetic analysis at the time of t-MDS diagnosis. 

## Conclusions

LC can be the first sign of a new leukemia diagnosis or the transformation of an existing hematologic condition (t-MDS in this case) to leukemia. We aim to raise awareness amongst dermatologists and internists as these patients may initially present to these specialists. Maintaining a high index of clinical suspicion, through a complete history and physical examination, is crucial in order to recognize rare cutaneous manifestations of common hematopoietic malignancies.
